# Endocrine‐disrupting chemicals and male reproductive health

**DOI:** 10.1002/rmb2.12326

**Published:** 2020-04-14

**Authors:** Aditi Sharma, Josephine Mollier, Richard W. K. Brocklesby, Charlotte Caves, Channa N. Jayasena, Suks Minhas

**Affiliations:** ^1^ Section of Investigative Medicine Faculty of Medicine Imperial College London London UK; ^2^ Imperial Centre for Andrology Imperial College Healthcare NHS Trust London UK

**Keywords:** endocrine‐disrupting chemicals, environmental, male infertility, semen quality, sperm

## Abstract

**Background:**

A number of different types of endocrine‐disrupting chemicals (EDCs) including bisphenol A, phthalates, pesticides, and other environmental chemicals have been shown to adversely impact upon male reproductive health. Understanding the potential effects of EDCs on male reproductive health may enable the development of novel treatments and early prevention of the effects of EDCs on male infertility and their potential long‐term sequelae. This review critically evaluates the research performed in this area and explores potential harmful effects of EDCs in animals and humans, including the possibility of trans‐generational transmission.

**Methods:**

A literature review was conducted using electronic databases using the following terms: ‘endocrine disrupt*’ OR ‘endocrine disruptors’ OR ‘endocrine disruptor chemicals’ OR ‘EDC’ AND ‘sperm*’ OR ‘spermatozoa’ OR ‘spermatozoon’ OR ‘male reproductive health’ OR’ male fertility’.

**Main findings:**

Several studies have shown that EDCs have a variety of pathophysiological effects. These include failure of spermatogenesis, embryonic development, the association with testicular cancer, and long‐term metabolic effects.

**Conclusions:**

Several studies observe correlations between chemical doses and at least one sperm parameter; however, such correlations are sometimes inconsistent between different studies. Mechanisms through which EDCs exert their pathophysiological effects have not yet been fully elucidated in human studies.

## 1 INTRODUCTION

A number of studies have reported a deterioration in sperm quality over the past 50 years,^1^, ^2^ and a variety of factors have been implicated in this decline, including environmental and lifestyle factors. Data from animal and human studies suggest that endocrine‐disrupting chemicals are associated in the etiopathogenesis with harmful effects on male reproductive health.^3^ Furthermore, recent meta‐analysis has suggested that EDCs not only have a deleterious effect on sperm quality but may also be associated with cryptorchidism, hypospadias, and testis cancer, the so‐called testicular dysgenesis syndrome.^4^

It has been postulated that environmental chemicals may affect the endocrine regulation of fertility since the 1920s.^5^ Over the past decade, many chemicals have been observed to have the ability to disrupt the endocrine system and male reproductive health. The European Union Scientific Committee of Toxicity, Ecotoxicity and Environment defines an endocrine‐disrupting chemical (EDC) as “an exogenous substance or mixture that alters function(s) of the endocrine system and consequently causes adverse health effects in an intact organism, or its progeny, or (sub)population”.^6^ Humans are exposed to these chemicals daily, as they are found ubiquitously in the environment and in everyday objects (Table 1). There have been many advances in our understanding of these chemicals and their potential impact on male reproductive health. The different types of EDCs discussed in this review are listed in Table 1. This review will critically explore current research linking the role of endocrine‐disrupting chemicals such as bisphenol A (BPA), insecticides, phthalates, and other common environmental chemicals with the decline of male reproductive health.

**Table 1:** Summary of postulated endocrine‐disrupting chemicals and their common use

Postulated EDC	Common uses/exposure
Bisphenol A	Manufacture of polycarbonate plastics, used in food packaging, water containers, dental sealants
Phthalates	Plasticizers, used in packaging, personal care products, industrial plastics, medical devices, pharmaceuticals.
Parabens (eg, butylparaben)	Preservative, found in food, cosmetics, toiletries, medications
Nonylphenol ethoxylates	Detergents, paint, pesticides, personal care products, plastics
Tributyltin chloride	Consumer goods and industrial products
Genistein	Soy derived products
Silver nanoparticles	Antibiotics, burn wound dressings, surgical devices, prosthetic bones
Perfluoroalkyl compounds	Carpets, textiles, paper
Triclosan	Personal care, household, industrial, and veterinary products
Octylphenol	Sewage, farm animals’ tissues grazed on sewage‐contaminated ground
Microcystin‐LR	Freshwater
Chlorotriazine herbicides (eg, atrazine)	Herbicide, ground water
Insecticides	Fresh produce, bioaccumulation in the environment
Glyphosate	Herbicide
Dichlorodiphenyltrichloroethane	Pesticide
Vinclozolin	Fungicide used in fruit and vegetables
Benzo[a]pyrene	Formed from incomplete combustion of organic material, for example, diesel exhaust, cigarette smoke, charcoal cooked food, cooking oil fumes, industrial waste by‐products
Polycyclic aromatic hydrocarbons	Environmental pollutant from incomplete combustion of coal, petrol, oil, and wood
Polybrominated diphenyl ethers	Flame retardants used in building materials, furnishings, electronics
Dioxins	By‐products of chlorine bleaching of pulp and paper, manufacture of certain pesticides, and incineration of medical waste and plastics

**Figure 1:**
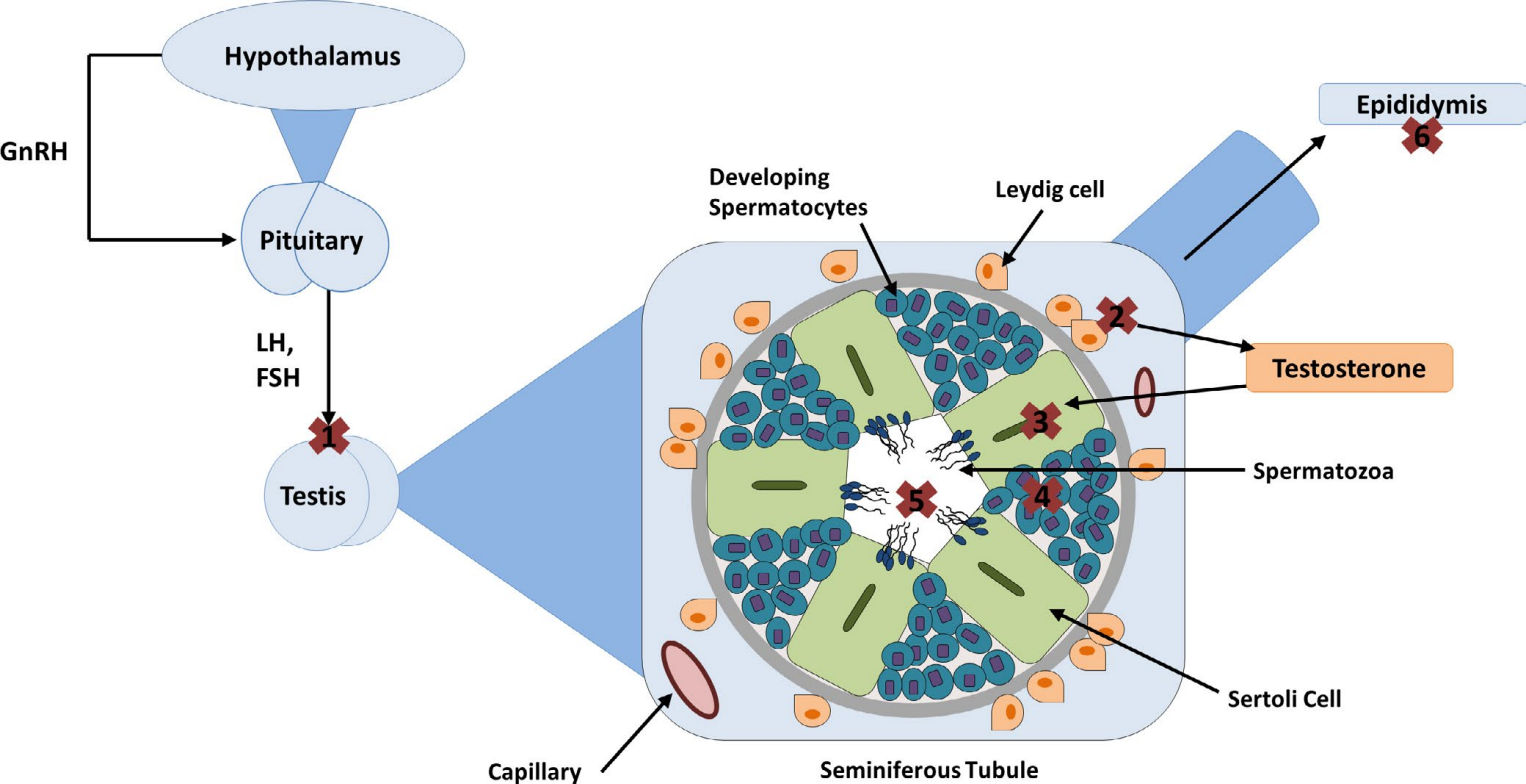
The main mechanisms by which EDCs disrupt sperm quality, denoted by crosses 1‐6. GnRH: gonadotropin‐releasing hormone, LH: luteinizing hormone, FSH: follicle‐stimulating hormone. (a) Disruption of testicular gonadotropin receptors, (b) disruption of Leydig cell steroidogenesis, (c) Sertoli cell damage, (d) inhibition of spermatocyte development, (e) disruption of mature sperm, 6: disruption of epididymal sperm modification

EDCs may inhibit the action of endocrine action at a receptor or cellular level.^3^ These chemicals act via a variety of mechanisms, such as mimicking endogenous hormones via an agonistic effect, blocking their action via an antagonistic effect, or interfering with metabolic activity. Sperm development and quality is under multiple levels of regulation, therefore be disrupted at many points. The hypothalamus produces gonadotropin‐releasing hormone (GnRH) which stimulates gonadotropin release; luteinizing hormone (LH), and follicle‐stimulating hormone (FSH) from the anterior pituitary gland, and this can be disrupted at any stage. Figure 1 summarizes the main sites for EDC action. Testicular effects include increased spermatocyte apoptosis via Sertoli cell damage^7^ or up‐regulation of apoptotic proteins.^8^ Sertoli cells nourish developing spermatocytes and absorb excess cytoplasm and maximize testosterone‐induced spermatogenesis. Failure of testosterone production in Leydig cells leads to failure of testosterone‐bound androgen receptor‐mediated gene transcription necessary for spermatogenesis. Some studies suggest that EDCs such as BPA inhibit ATP production,^9^ perhaps by disrupting mitochondria,^10^ impairing sperm motility. An abnormal hormonal milieu caused by EDCs may lead to aneuploidy in sperm and potential transgenerational effects. However, many of these postulated mechanisms need to be substantiated in clinical studies to define the exact mechanisms by which EDCs exert their effects in humans.

## 2 MATERIALS AND METHODS

### 2.1 Search and selection

A search of the electronic databases Embase, MEDLINE, and PubMed, was conducted during January 2019. All studies identified using search terms up until January 2019 were considered for inclusion to the study. Databases were searched using the following terms: ‘endocrine disrupt*’ OR ‘endocrine disruptors’ OR ‘endocrine disruptor chemicals’ OR ‘EDC’ AND ‘sperm*’ OR ‘spermatozoa’ OR ‘spermatozoon’ OR ‘male reproductive health’ OR’ male fertility’. Identified studies were excluded if the abstracts were not in English language. Both animal and human studies were reviewed.

### 2.2 Data extraction

Study titles and abstracts were initially screened before full‐text review was completed in duplicate by two study investigators (RKB, CC). Discrepancies were dealt with by consensus discussion.

## 3 ANIMAL STUDIES

### 3.1 Bisphenol A

Bisphenol A has been shown to have various effects on sperm quality in animals, as summarized in Table 2. Many studies suggest that BPA compromises DNA integrity. DNA integrity is a prerequisite for embryonic development. DNA damage can lead to failure of embryogenesis and early miscarriage, or it can lead offspring with significant birth defects. Numerous animal studies have demonstrated that BPA affects sperm parameters, hormone levels, and fertility, but often findings are not reproducible and mechanisms for its action are not well understood. BPA may have a combination of effects on factors involved in spermiogenesis, which may contribute to a reduction in fertility potential.

**Table 2:** Summary of the effects of BPA observed in animal studies

Species	Observations	Study
Mouse	Decline in daily sperm production Reduced motility	Tainaka et al^79^
Mouse	Reduced DNA integrity Reduced acrosome integrity	Kalb et al^80^
Mouse	Decreased sperm motility Reduced membrane and acrosomal integrity Decreased intracellular ATP and mitochondrial enzymes	Rahman et al^10^
Mouse	Seminiferous tubule damage Mitotic arrest at spermatogonia stage Increased apoptotic germ cells Increased chromosome fragments	Xie et al^81^
Mouse	Transient inhibition of CatSper, acrosomal reaction, and motility.	Wang et al^82^
Rat	Persistence of DNA strand breaks during pachytene. Increased spermatocyte apoptosis	Liu et al^83^
Rat	Reduced daily sperm production Reduced motility Reduced DNA integrity Reduced acrosome integrity	Tiwari et al^84^
Sterlet	Decreased sperm motility and velocity in vitro Atypical flagella and reduced beat frequency Dose‐dependent effect on intracellular ATP content	Hulak et al^9^
Fish	Decreased sperm motility Reduced fertilizing ability	Singh et al^85^
Rare minnow	Increased apoptotic germ cells Increased chromosome fragments Increased mitochondrial apoptosis proteins, for example, Bcl2 and caspase‐9	Zhang et al^8^

### 3.2 Pesticides

Some studies have also found associations between insecticides, herbicides, and fungicides with semen quality. Daily deltamethrin, a pyrethroid insecticide, has been shown to decrease sperm quantity, motility, and vitality in rats.^11^ Testosterone and inhibin B levels were also decreased, suggesting an underlying primary testicular dysfunction. Altered seminiferous tubules, sloughed germ cells, and vacuolization of Sertoli cells were also observed on testicular histology.

Glyphosate, a herbicide, decreased sperm production in mice whose mothers were exposed to the chemical.^12^ Testosterone concentration was decreased at puberty, with a dose‐associated relationship. Testicular histology demonstrated decreased elongated spermatids and seminiferous tubule degeneration. Methoxychlor has also been shown to result in a reduction in proteins required for steroidogenesis, including StAR protein, 3‐beta‐hydroxysteroid dehydrogenase (3‐beta‐HSD), and 17‐beta‐HSD in rats.^13^ This may explain the decreased testosterone concentrations in this animal model.

Vinclozolin, a fungicide, has been shown to reduce testosterone production and spermatozoa after human chorionic gonadotropin (hCG) stimulation, but no difference was seen in the mRNA levels of StAR protein, p450scc, or other proteins involved in steroidogenesis. When given with genistein, another EDC, the effect was compensated for by a thus far unknown mechanism.^14^ Fenvalerate is an insecticide which has been found to decrease sperm count and both serum and testicular testosterone, and increase apoptosis of germ cells in the seminiferous tubules.^15^

An in vitro study has investigated the effect of atrazine (ATZ), a chlorotriazine herbicide and its metabolite, diaminochlorotriazine (DACT).^16^ 0.1, 1, and 10 μM of ATZ were found to increase the proportion of dead sperm without dose dependency, and the proportion of sperm with damaged membranes in a dose‐associated manner. Interestingly, only sperm from the tail of the epididymis was affected at all doses, sperm from the epididymal body was affected at high doses, and sperm from the head of the epididymis was unaffected. After 2 and 4 hours of incubation, the proportion of sperm entering pseudo‐acrosomal reaction (whereby the acrosomal reaction is induced without a known stimulation) was increased, with all doses of ATZ compared to controls.

In another study, intraperitoneal ATZ was given to Fischer rats twice a week for 60 days. Reduced sperm motility was found, but there was an increase in sperm number.^17^ Testicular histology revealed irregular Leydig cells and Sertoli cells, as well as cell clusters with spermatocytes. Results from these studies suggest that these chemicals also have a direct toxic cellular effect leading to testicular damage. A reason for the increased sperm count may be explained by the undamaged parts of the testis being exposed to paradoxically larger quantities of testosterone and thus producing higher numbers of sperm, which do not gain motility.

Many studies have shown that various pesticides decrease testosterone levels. Testosterone is required for the final stages of sperm maturation, so a decrease in intra‐testicular testosterone is likely to impair fertility. However, the mechanisms for this are poorly understood. It is also unclear if all pesticides studied decrease testosterone and fertility in the same manner.

### 3.3 Phthalates

Phthalates are also commonly studied as potential EDC. The effects of di(2‐ethylhexyl) phthalate (DEHP) have been studied in mice and shown to cause a 30% reduction in daily sperm production, a 70% reduction in epididymal sperm count, and a 20% reduction in sperm viability.^18^ Intratesticular testosterone was decreased, and the FSH and LH receptors were down‐regulated. Another study confirmed reduced sperm count and seminiferous tubule atrophy on histology.^19^ Reduced gonadotropin signaling may result in reduced steroidogenesis, which may lead to the observed seminiferous tubule depletion. However, it is unclear as to the cause of the decreased receptor expression, although serum testosterone was not measured. Another study reported decreased sperm count in males born to exposed mice, but only in c57BL/6J strain mice,^20^ although FVB/N mice were not affected. Increased hypermethylated gene promoters were found in the c57BL/6J strain. This suggests that there may be genetic or epigenetic predisposition to the effects of DEHP in mice. However, this study used 300 mg/kg/day dose of DEHP, which is 150 000× the recommended levels advocated by the Environment Protection Agency, and it is unclear if lower and more “physiological” daily exposure would produce the same effect.

### 3.4 Parabens

Parabens have been shown to lead to a reduction and disappearance of the seminiferous tubule lumen with very few germ cells seen in the tubules 3 hours after a single administration of the chemical.^21^ This effect was time‐dependent. A TUNEL assay also showed an increased number of apoptotic germ cells, but the mechanism of this is not known. More research into the adverse endocrine effect of parabens is required.

### 3.5 Combustion products

Chemicals formed from incomplete combustion of organic materials may also have endocrine‐disrupting effects. Benzo[a]pyrene (B[a]p) has been shown to decrease serum testosterone and increase apoptotic germ cells in a dose‐dependent manner.^22^ Reduced sperm motility was observed at all doses, and acrosomal integrity was reduced. hCG‐stimulated testosterone production was decreased in cells exposed to B[a]P, and this was most remarkable with higher doses. Long‐term exposure resulted in reduced expression of StAR protein and 3‐β‐HSD enzymes, which are both crucial for steroidogenesis. P450scc enzymes were increased, as a possible compensatory mechanism for producing pregnenolone with little cholesterol available. Sperm hyperactivity and abnormal acrosome reactions were observed due to exposure to 25 µg/mL of B[a]p in an in vitro study.^23^ It is thought that B[a]P is capable of increasing intracellular calcium via tyrosine phosphorylation. Polybrominated compounds have been found to cause decreased number and viability of spermatozoa.^24^ Expression of StAR protein and cytochrome p450scc enzymes, as well as reduced 3‐β‐HSD and 17‐3‐β‐HSD activity, again suggests impaired steroidogenesis. Interestingly, an altered thyroid status was also observed likely due to impaired steroidogenesis from down‐regulation of steroidogenic factor 1 (SF‐1).

### 3.6 Dioxins

Among the dioxins, 2,3,7,8‐tetrachlorodibenzo‐*p*‐dioxin (TCDD) is the most toxic environmental contaminant in animal studies. TCDD can bind and activate the aryl hydrocarbon receptor (AhR). which is a ligand‐activated transcription factor expressed in many human tissues^25^ Gestational administration of TCDD reduced ejaculated and epididymal sperm count in male rat offsprings without a reduction in serum testosterone or androgen receptor (AR) levels.^26^ Sex accessory gland weights in the rat offsprings were also reduced. The mechanisms underlying reduced epididymal sperm counts are unknown; however, AhR activation and/or an effect on epididymal function is a potential target.^25^, ^27^ In addition, prenatal TCDD administration on gestational day 15 (at 0.064‐1 µg/kg) demasculinized and feminized morphology of Holtzman male rat offsprings.^26^ Abnormal sperm parameters and sperm viability were also reported^28^, ^29^ Spermatogenic markers (such as acid phosphatase, alkaline phosphatase, lactate dehydrogenase) were significantly altered in the testes. Testicular histology showed necrotic degeneration in dioxin exposed mice in comparison with controls.^29^ Furthermore, reduced expression of steroidogenic markers such as StAR protein, 3‐β‐HSD, and 17‐3‐β‐HSD was also reported suggestive of impaired steroidogenesis^28^ with reduced circulating levels of both testosterone and dihydrotestosterone (DHT) in adult rats exposed to high concentrations of TCDD^30^ Furthermore, TCDD suppressed the expression of glutamic acid decarboxylase 67, an enzyme involved in GABA synthesis in the brain of gestationally exposed rats, potentially preventing the perinatal surges of LH and testosterone compromising sperm counts.^31^ Results from these studies suggest both an adverse endocrine effect as well as direct testicular toxicity.

### 3.7 Other common household chemicals

Oral tributyltin chloride has been shown to decrease sperm count in mice and sloughing of germ cells by an unknown mechanism.^32^ High‐dose nonylphenol metabolites were associated with abnormal sperm and decreased sperm motility in rats after 2 hours,^33^ among other behavioral abnormalities in learning and memory. Sperm viability was decreased at all doses, and even a 1mg dose caused decreased motility after 4 and 6 hours of exposure. It has also been found to cause spermatid necrosis and seminiferous tubule dilation at the 250 mg dose in ducks.^34^ However, sperm count and fertility rate were not significantly affected in this study. Subcutaneous octylphenol increased the number of abnormal sperm but there were no differences in sperm concentration or semen volume. Silver nanoparticles have also been studied in rats, and these rats were found to have reduced acrosome reactions, plasma membrane integrity and mitochondrial activity, as well as an increased number of abnormal sperm.^35^ No changes in serum FSH, LH, testosterone, or estrogen were found, suggesting a direct effect on sperm. Microcystin‐LR has also been investigated and was shown to decrease numbers of sperm, motility, and rate of sperm abnormalities in frogs.^36^ Serum testosterone was decreased, and estrogen was increased.

## 4 HUMAN STUDIES

### 4.1 Bisphenol A

Observational studies have found associations between BPA and infertility. A study analyzing semen samples from 191 men attending an assisted reproductive clinic found that 93% had BPA in their semen.^37^ Seminal BPA was negatively associated with sperm count and motility. However, another observational study of 364 men found no correlation between BPA concentration and sperm count, motility, or semen volume.^38^ This study, however, did find that BPA was negatively associated with sperm count in obese men. This may be due to slower metabolism of BPA in obesity, thereby amplifying its effects. A further study found a significant increase in BPA levels in a group of sub‐fertile men, and an association between urinary BPA and reduced semen quality with at least one semen parameter below WHO reference values.^39^ Similarly with animal studies, the effects of BPA were often not reproducible, but it was evident that BPA can lead to infertility.

### 4.2 Pesticides

The effect of pesticides and insecticides has also been investigated in humans, although not as extensively. P,p'‐dichlorodiphenyldichloroethylene (p,p'‐DDE) is a metabolite of dichlorodiphenyltrichloroethane (DDT) which is a pesticide. It is no longer used in developed countries, but can accumulate in adipose tissue and is poorly excreted.^40^ In vitro*,* high doses affected sperm viability in conditions that mimicked the female reproductive tract, and CatSper inhibitors reversed this effect, suggesting that it over stimulates Catsper. Catsper is the calcium channel required for sperm capacitation and is therefore essential for sperm motility.

Dialkyl phosphates, metabolites of organophosphorus insecticides, were found in high frequency in the semen of infertile men. Exposed men were observed to have higher than expected amounts of aneuploid sperm which could lead to miscarriage.^41^ These findings are not consistent with animal studies of pesticides, which suggested that they reduce testosterone production.

### 4.3 Phthalates

Phthalates have also been investigated in humans via observational studies. A meta‐analysis showed that abnormal sperm quality was significantly associated with phthalates.^42^ Further studies have shown a negative correlation between phthalate metabolites and abnormal sperm morphology.^43^ Some metabolites were also negatively associated with acrosin activity and INSL3, a Leydig cell function marker. Phthalates were also associated with increased LH, decreased testosterone, and increased estradiol production by Leydig cells, as well as increased Leydig cell numbers.^44^ A Chinese study also observed decreased serum free testosterone in phthalate exposed factory workers compared to a control group.^45^ However, another study conducted in 425 men attending a fertility clinic found an association between urinary phthalate metabolites and decreased estradiol, decreased free androgen index, and increased testosterone:estradiol ratio.^46^ However, other studies found no association between serum phthalate metabolites and estradiol or testosterone, but a dose‐dependent increase in prolactin in those with high serum phthalate.^47^ A placebo‐controlled trial in 26 men exposed to a cream containing the phthalate metabolites for a week; then, exposure to a cream without DEP showed changes in estradiol and inhibin B levels but these changes were not consistent.^48^ However, the sample size and study duration may not have been adequate to draw firm conclusions. Further longitudinal studies are needed to fully elucidate the effects of EDC. However, the unknown long‐term cumulative effects of EDCs present ethical challenges.

### 4.4 Flame retardants

The effects of polybrominated diphenyl ethers on reproductive function have also been investigated in humans. In sperm samples taken from men attending fertility clinics, sperm motility was negatively correlated with concentrations of certain subtypes of these chemicals, but not others.^49^ No association was found with sperm density or abnormal morphology. Brominated flame retardants have also been associated with increased risk of infertility by factor of 7.22% and 33% lower sperm motility in studies.^50^ Brominated compounds may affect the balance of reproductive hormones, causing impaired fertility, but again the underlying pathophysiological mechanisms are not yet fully understood.

### 4.5 Dioxins

Human exposure to dioxins is mainly through consumption of contaminated food especially high‐fat foods such as milk, cheese, meat, and breast milk.^51^, ^52^ Epidemiological studies suggest that exposure to TCDD can lower the male/female ratio of offspring.^53^ A study reported semen quality in men stratified on the basis of their age at time of acute exposure to high levels of TCDD. Decreased sperm concentrations were observed in men between 1 and 9 years of age at the time of exposure with no effects in adults^54^ suggestive of a critical window for the developing reproductive tract and potential epigenetic modifications. In addition, a negative and significant correlation was observed between testosterone levels and the levels of seventeen 2,3,7,8‐substituted congeners of polychlorinated dibenzo‐p‐dioxins (PCDDs) and polychlorinated dibenzofurans (PCDFs) and four non‐ortho polychlorinated biphenyls (PCBs) in 42 men who resided near a dioxin‐contaminated area in Vietnam.^55^

### 4.6 Other common household chemicals

Perfluoroalkyl compounds groups have been associated with a lower percentage of sperm with normal morphology and sperm count, but non‐significant trends were found when the compounds were considered separately. Triclosan, a household chemical used in toothpaste, has been shown to be negatively correlated with sperm concentration and count, normal morphology proportion, and progressive sperm in infertile men.^56^ Another study found a negative association between triclosan and inhibin levels, as well as positive associations with LH, but the mechanisms for decreased sperm quality have not been elucidated.^56^

Parabens have also been investigated in humans. In a study analyzing semen quality from 315 infertile patients, there was a significant association between urinary paraben concentrations and increased percentage of sperm with abnormal morphology, as well as decreased motility, but no change in estradiol levels.^57^ This suggests that there was no effect on steroidogenesis, but the mechanisms by which sperm quality was affected remain unclear.

### 4.7 In utero exposure, testicular dysgenesis, and testicular cancer

EDCs exposure in utero may lead to developmental abnormalities in the male, which may result in reproductive abnormalities in adult life and includes cryptorchidism, hypospadias, poor semen quality, and a predisposition to testicular germ cell cancers—the so‐called testicular dysgenesis syndrome (TDS).^4^ A number of authors have postulated that the etiology of TDS is the result of inhibition of androgen action on fetal development leading to Sertoli and Leydig cell dysfunction.

The effects of EDCs may be the result of direct or epigenetic effects, which alter fetal masculinization and have been illustrated in both animal studies and observational human studies. The incidence of TC has risen dramatically in the past few decades,^58^ and it is has been suggested that reduced sperm quality, testicular cancer, and testicular dysgenesis may have a common origin in utero (see below).

### 4.8 Sperm quality

In utero exposure to BPA can cause decreased sperm quality, whereby exposed mice produced sperm with significantly decreased sperm membrane integrity, motility, and in vitro penetration rates.^59^ Seminiferous tubule disruption and decreased germ cells within tubules,^7^ occurred without a dose‐dependent effect. Electron microscopy showed lesions in endoplasmic reticulum (ER) of Sertoli and Leydig cells, and complete absence of Sertoli cells when BPA was combined with diethylstilbestrol, another endocrine‐disrupting chemical. Western blot showed increased CHOP protein, which is a marker of ER stress.

Cypermethrin, a pyrethroid insecticide, was shown to have no effect on amount of apoptotic cells or Leydig cells,^32^ but serum testosterone and number of spermatozoa were decreased in mice whose mothers were exposed to this chemical. Levels of testicular cytochrome P450 side‐chain cleavage enzymes (p450scc) were decreased. It has also been shown to cause atrophy of the seminiferous tubules with decreased spermatocytes and delayed testicular decent ^60^ in male mice born from exposed mothers. These findings have been extrapolated to human studies and may account for the observed decline in sperm quality in men over the last 50 years.^1^, ^2^

### 4.9 Testicular dysgenesis

A meta‐analysis suggested that anogenital distance was associated with testicular abnormalities and may indicate a mechanism by which EDCs affect reproductive health, as it is a sensitive biomarker of prenatal androgen action.^3^ Similarly, another study suggested that first‐trimester phthalate exposure leads to a shorter AGD,^61^ but some studies found no correlation. Exposure to dibutyl phthalate in particular led to disrupted male differentiation and reduced AGD.^62^ However, study designs were not consistent, and some confounding factors such as weight and body length were not accounted for. A further meta‐analysis assessed the effects of exposure to EDCs in utero or infancy in 33 studies.^63^ In particular, exposure to p,p‐DDE was related to an increased risk of cryptorchidism, hypospadias, low sperm count, and testicular cancer.

### 4.10 Testicular cancer

A case‐control study has shown that the use of pesticides and other possible exposure to EDCs were more common among case subjects, but no dose‐dependent effect was found. No significant association with TC was found when investigating maternal exposure to EDCs.^64^ However, it is hard to quantify exposure in observational studies.

Organo‐chlorine pesticide levels in mothers have been shown to be correlated with increased risk of testicular germ cell cancer compared to controls.^64^ However, organo‐chlorine levels were measured retrospectively so it is unclear if their sons had increased exposure in utero. Estrogenic substances can be carcinogenic, and estrogen agonism may have a pathophysiological role in testicular dysgenesis and development of testicular cancer, but the exact mechanisms remain unclear.

### 4.11 Other in utero effects

A further study demonstrated that offspring exposed to BPA had reduced sperm counts.^65^ There was also decreased sperm count. Testis, seminiferous tubules, and prostate weight were unaffected. Studies have also found that BPA may be associated with intrauterine growth restriction in mice, resulting to reduced birth weight and this may be via altered methylation of the Igf2 gene, which is known to be important to fetal growth.^66^ Only the metabolic effects of this epigenetic gene modification were observed in F2; body weight was not significantly different to controls. Body weight was unaffected in most other studies, as shown in a recent meta‐analysis.^67^

A meta‐analysis found that “possible” and “probable” exposure to EDCs during pregnancy was associated with an increased risk of low birth rate, length of gestation, and preterm delivery. Phthalates, metals, alkylphenol compounds, brominated flame retardants, polycyclic aromatic hydrocarbons were found to have the greatest effect.^68^ Exposure was defined by women exposed to EDCs in their work, that is, agriculture workers and hairdressers. However, many workers were exposed to a cocktail of EDCs so it is unclear as to exactly which chemicals had these effects. Also, it is unclear if workers were actually exposed to the expected EDCs.

A link between gestational exposure to phthalates and autism has been also been suggested in a review of 7 studies.^69^ Brain development is vulnerable to teratogens from the 4‐week gestation to delivery, and they may be most vulnerable when the blood‐brain barrier is not fully formed. However, mechanisms for this effect are poorly understood.

## 5 METABOLIC SYNDROME

Metabolic syndrome is a cluster of 5 characteristics including centripetal obesity, hypertension, high blood glucose, high serum triglycerides, and low serum high‐density lipoprotein (HDL). It predisposes to type 2 diabetes and cardiovascular disease. A recent meta‐analysis of 420 studies has shown a positive association between urine and serum BPA and type 2 diabetes mellitus.^70^ BPA may bind to pancreatic islet cells and impair insulin and glycogen secretion. It can also act at insulin‐sensitive tissues such as muscle, liver, and adipose tissue resulting in an insulin‐resistant state, leading to promotion of diabetes and metabolic disorders such as the metabolic syndrome. This effect may be greater during the rapid growth phase, that is, in utero exposure or childhood, although, the mechanistic basis in humans is understood. There is also potential publication bias. A meta‐analysis of 31 studies analyzing early‐life exposure to di(2‐ethylhexyl) phthalate found increased fat weight and elevated triglycerides in rats,^71^ but whole body weight was not affected. Phthalate exposure has also been shown to have a positive correlation with metabolic dysregulation, leading to increased waist circumference, insulin resistance, and obesity, key components of the metabolic syndrome.^72^

## 6 TRANSGENERATIONAL EFFECTS OF EDCS

There is evidence that some EDCs may not only have effects on those exposed, but also effects that persist through generations. This may be via epigenetic changes, which may not manifest until F2‐4 generations. EDCs may also cause increased DNA fragmentation and aneuploidy, which may lead to miscarriage and genetic abnormalities in offspring. Male mice born to dams exposed to daily oral BPA, from gestational day 12 to postnatal day 21, had reduced fertility when mated with unexposed females.^73^ This effect persisted for 3 generations. Immunohistochemistry of the testis revealed that steroid receptor expression was decreased in F1‐F3. Studies in mice have shown that BPA caused altered social recognition until the F4 generation, via altered expression of Oxt and Avp genes.^74^ Another study has shown that BPA leads to an obese phenotype in the F3 generation, but not F1 and 2. Genes associated with obesity were found to have altered methylation in F3 generation's sperm, but this was not assessed in F1 and F2.

Rats exposed to daily butyl benzyl phthalate were observed over two generations. At the doses above 200 mg/kg, body weight lower epididymal weights were observed. At the high dose, 400 mg/kg body weight, the F1 generation had diffuse atrophy of the seminiferous tubules and decreased spermatozoa, with higher doses producing Leydig cell hyperplasia, small testis, low seminal vesicle weights, and partial aplasia or aplasia of the epididymis. The F2 generation demonstrated increased feminization as indicated by a reduction in anogenital distance. Another study found a relationship between urinary phthalates and Y chromosome‐bearing sperm,^75^ but mechanisms of this are unknown.

Cypermethrin, an insecticide, has been studied over 2 generations, and not only did it affect sperm parameters in the male mice born to exposed mothers, but fertility was decreased and reduced fetal weight was observed in the F2 generation, and some mice had malformations such as underdeveloped bodies and deformed limbs.

The underlying mechanism for these transgenerational effects may be explained by the negative effects of EDCs on sperm DNA integrity and increased aneuploidy rates observed in human studies. In one study, urinary levels of pyrethroids were associated with sperm DNA damage.^76^ In a further human study, urinary concentrations of pyrethroid metabolites were associated with increased sperm aneuploidy rates.^77^

In humans, phthalates have also be associated with behavioral changes in children, including increased aggression and inattention.^78^

## 7 LIMITATIONS OF STUDIES

There are a number of limitations on the evidence linking EDCs and their effects on male reproductive health. Many animal studies involve the use of artificial female reproductive tracts, which may not be representative of natural mating and animal models. There is also limited applicability of animal studies to humans as hormone signaling often varies between species. This is supported by a study that observed varying responses to chemicals depending on the strain of mice used.^20^ While animal studies are useful to study the histological effects of chemicals on the testes, testicular histology is rarely obtained in human studies.

A variety of doses of chemicals were used in studies with many studies observing the effect of a large dose over a short period of time. However, many chemicals are only present in small doses in the environment with exposure over a longer period of time. Therefore, further research is required to determine the adverse effects of dosages that humans are exposed to in the environment.

Multiple animal studies have demonstrated dose‐dependent adverse effects of EDCs on spermatogenesis and steroidogenesis. Longer, cumulative doses may be suggestive of an irreversible dysfunction with trans‐generational epigenetic effects. Furthermore, spermatogenesis is a remarkably complex process, with many regulating factors for its dysfunction such as developmental age of exposure, route of administration, dose‐response, short‐ and long‐term effects. Yet, very few longitudinal studies have been performed in humans to conclude the possible mid‐term to long‐term effects of exposure to low doses or epigenetic effects on future generations. Most studies in humans are observational studies, using data from infertility clinics. These patients may be more susceptible to the effects of EDCs due to confounding variables, for example, hormone dysfunction, genetic susceptibility and hence may not be representative of the general population. Additionally, while a variety of sperm parameters were used to assess sperm quality, but often pregnancy or live birth rates were not documented. Larger powered studies reporting on live birth rates are required to objectively quantify the absolute effects of EDCs on male fertility.

The trans‐generational effects of these chemicals on fertility and congenital abnormalities in offspring are under‐reported. Also, while synergistic effects of chemical combinations are plausible, they have as yet to be studied. Furthermore, a number of meta‐analyses have found inconsistent results between studies, with significant heterogeneity and risk of bias with regard to EDCs and their differing effects on male reproductive health.

## 8 CONCLUSIONS

Evidence suggests that a growing number of environmental chemicals adversely affect sperm quality and reproductive health in men. Although many studies and in particular animal studies observe correlations between chemical doses and at least one sperm parameter, such correlations are often inconsistent between different studies. It is likely that one EDC may act via a variety of different mechanisms, but these have not yet been fully elucidated in human studies, and further research is required to determine the mechanisms by which EDCs exert their pathophysiological effects. There is a need for well‐conducted, longitudinal, multi‐center studies in different geographical populations. Finally, the full, transgenerational effects of these chemicals need to be investigated to determine the potential of EDCs to impart cumulative adverse effects on male reproductive health in successive generations of the population.

## DISCLOSURES

*Conflict of interest*: Aditi Sharma, Josephine Mollier, Richard K Brocklesby, Charlotte Caves, Channa N. Jayasena, and Suks Minhas have stated explicitly that there are no conflicts of interest in connection with this article. CNJ and SM have previously received research funding from LogixX Pharma as stated in COI forms. *Human rights statements and informed consent/Animal studies*: This article does not contain any studies with human and animal subjects performed by the any of the authors. *Approval by ethics committee: *No ethical approval was needed for this review article.
